# 2-[(4-Methyl­phen­yl)sulfan­yl]aniline

**DOI:** 10.1107/S1600536811002820

**Published:** 2011-01-26

**Authors:** Richard Betz, Thomas Gerber, Henk Schalekamp

**Affiliations:** a Nelson Mandela Metropolitan University, Summerstrand Campus, Department of Chemistry, University Way, Summerstrand, PO Box 77000, Port Elizabeth 6031, South Africa

## Abstract

The least-squares planes defined by the aromatic moieties in the title aniline derivative, C_13_H_13_NS, are nearly perpendicular to each other, forming a dihedral angle of 87.80 (7)°. Apart from a weak intramolecular N—H⋯S hydrogen bond, a co-operative set of N—H⋯N hydrogen bonds present in the crystal structure leads to the formation of tetra­meric units.

## Related literature

For structures of aniline derivatives bearing an S atom in the *ortho* position to their respective amino group(s), see: Yuan *et al.* (2008 ▶); Sellmann *et al.* (1999 ▶); Heinisch *et al.* (1999 ▶). For graph-set analysis of hydrogen bonds, see: Etter *et al.* (1990 ▶); Bernstein *et al.* (1995 ▶).
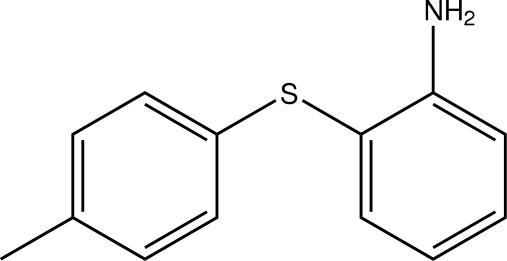


## Experimental

### 

#### Crystal data

C_13_H_13_NS
*M* *_r_* = 215.30
Tetragonal, 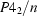

*a* = 17.8881 (7) Å
*c* = 7.2129 (3) Å
*V* = 2308.0 (2) Å^3^
*Z* = 8
Mo *K*α radiation
μ = 0.25 mm^−1^
*T* = 200 K
0.55 × 0.39 × 0.26 mm

#### Data collection

Bruker APEXII CCD diffractometer
10035 measured reflections
2748 independent reflections
2216 reflections with *I* > 2σ(*I*)
*R*_int_ = 0.055

#### Refinement

*R*[*F* ^2^ > 2σ(*F* ^2^)] = 0.075
*wR*(*F* ^2^) = 0.147
*S* = 1.20
2748 reflections
142 parameters
H atoms treated by a mixture of independent and constrained refinement
Δρ_max_ = 0.32 e Å^−3^
Δρ_min_ = −0.35 e Å^−3^

### 

Data collection: *APEX2* (Bruker, 2010 ▶); cell refinement: *SAINT* (Bruker, 2010 ▶); data reduction: *SAINT*; program(s) used to solve structure: *SHELXS97* (Sheldrick, 2008 ▶); program(s) used to refine structure: *SHELXL97* (Sheldrick, 2008 ▶); molecular graphics: *ORTEP-3* (Farrugia, 1997 ▶) and *Mercury* (Macrae *et al.*, 2006 ▶); software used to prepare material for publication: *SHELXL97* and *PLATON* (Spek, 2009 ▶).

## Supplementary Material

Crystal structure: contains datablocks I, global. DOI: 10.1107/S1600536811002820/tk2711sup1.cif

Structure factors: contains datablocks I. DOI: 10.1107/S1600536811002820/tk2711Isup2.hkl

Additional supplementary materials:  crystallographic information; 3D view; checkCIF report

## Figures and Tables

**Table 1:** Hydrogen-bond geometry (Å, °)

*D*—H⋯*A*	*D*—H	H⋯*A*	*D*⋯*A*	*D*—H⋯*A*
N1—H71⋯N1^i^	0.90 (3)	2.19 (4)	3.083 (3)	170 (3)
N1—H72⋯S1	0.81 (3)	2.60 (3)	3.032 (3)	115 (3)

Symmetry code: (i) 
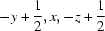
.

**Fig. 1.**
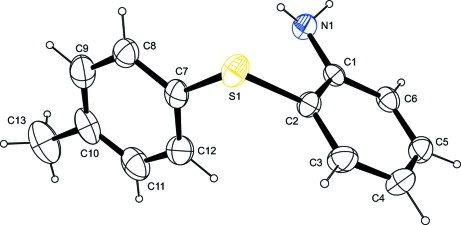
The molecular structure of the title compound, with atom labels and anisotropic displacement ellipsoids drawn at 50% probability level.

**Fig. 2.**
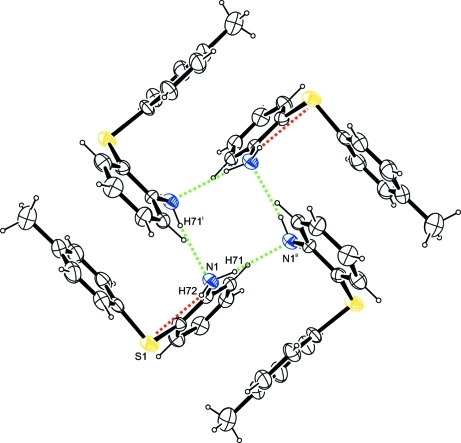
Hydrogen bonds in the title compound, viewed along [0 0 1]. The N–H···N contacts are illustrated in green, N–H···S contacts are illustrated in red. Symmetry operators: i y, -x + 1/2, -z + 1/2; ii -y + 1/2, x, -z + 1/2.

**Fig. 3.**
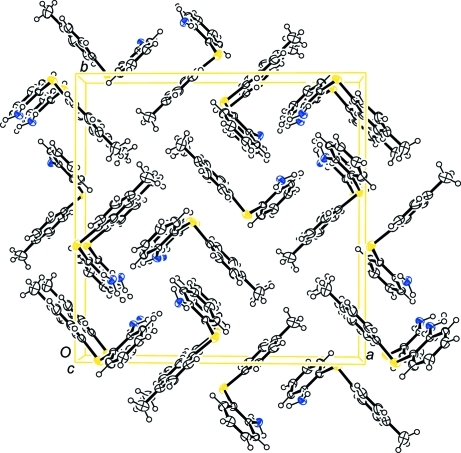
Molecular packing of the title compound, viewed along [-1 0 0] (anisotropic displacement ellipsoids drawn at 50% probability level).

## supplementary crystallographic information

### Comment

2-(*p*-Tolylthio)benzenamine is a derivative of aniline bearing a *para*-methylbenzene sulfide moiety in a position *ortho* to its amino group. Given its *N,S* set of donor atoms, it can act as a monodentate via either donor or as a bidentate ligand forming a five-membered chelate ring. The possibility to coordinate it as a purely neutral or, upon deprotonation, as an anionic ligand adds to its versatility. In our continued efforts to elucidate the coordination behaviour of nitrogen- and sulfur-containing ligands, it seemed of interest to determine the structure of the free ligand to enable comparative studies with related structures (Yuan *et al.*, 2008; Sellmann *et al.*, 1999; Heinisch *et al.*, 1999).

The least-squares planes defined by the two aromatic moieties in the molecule are orientated nearly perpendicular to each other; they enclose an angle of 87.80 (7)°. The C2–S1–C7 angle is 103.21 (12) ° (Fig. 1).

In the crystal structure, two different sets of hydrogen bonds can be observed, Table 1. While one of the hydrogen atoms of the amino group forms an intramolecular hydrogen bond to the sulfur atom, the remaining hydrogen atom of the NH_2_ group participates in a cooperative system of hydrogen bonds. The latter give rise to the formation of tetrameric units. In terms of graph-set analysis (Etter *et al.*, 1990; Bernstein *et al.*, 1995), the descriptor for the intramolecular interaction is *S*(5) while the cooperative system of hydrogen bonds necessitates a *C*^1^_1_(2) descriptor. The tetramer has a hydrophilic core which is shielded by the lipophilic parts of the molecules (Fig. 2). The closest distance between two aromatic systems is 4.0711 (16) Å. The molecular packing of the compound is shown in Fig. 3.

### Experimental

The structural analysis was performed on a sample taken from a commercially obtained (Sigma Aldrich) batch of the title compound.

### Refinement

H-atoms were placed in calculated positions (C—H 0.95 Å for aromatic C-atoms, C—H 0.98 Å for the methyl group) and were included in the refinement in the riding model approximation, with *U*(H) set to 1.2*U*_eq_(C) for aromatic carbon atoms and 1.5*U*_eq_(C) for the methyl group. The H-atoms of the amino group were located from a difference Fourier map and refined with *U*(H) set to 1.5*U*_eq_(N).

### Crystal data

**Table:**

C_13_H_13_NS	*D*_x_ = 1.239 Mg m^−^^3^
*M**_r_* = 215.30	Mo *K*α radiation, λ = 0.71073 Å
Tetragonal, *P*4_2_/*n*	Cell parameters from 5143 reflections
Hall symbol: -P 4bc	θ = 3.1–28.2°
*a* = 17.8881 (7) Å	µ = 0.25 mm^−^^1^
*c* = 7.2129 (3) Å	*T* = 200 K
*V* = 2308.0 (2) Å^3^	Block, colourless
*Z* = 8	0.55 × 0.39 × 0.26 mm
*F*(000) = 912	

### Data collection

**Table:**

Bruker APEXII CCD diffractometer	2216 reflections with *I* > 2σ(*I*)
Radiation source: fine-focus sealed tube	*R*_int_ = 0.055
graphite	θ_max_ = 28.0°, θ_min_ = 3.1°
φ and ω scans	*h* = −20→23
10035 measured reflections	*k* = −18→23
2748 independent reflections	*l* = −7→9

### Refinement

**Table:**

Refinement on *F*^2^	Primary atom site location: structure-invariant direct methods
Least-squares matrix: full	Secondary atom site location: difference Fourier map
*R*[*F*^2^ > 2σ(*F*^2^)] = 0.075	Hydrogen site location: inferred from neighbouring sites
*wR*(*F*^2^) = 0.147	H atoms treated by a mixture of independent and constrained refinement
*S* = 1.20	*w* = 1/[σ^2^(*F*_o_^2^) + (0.031*P*)^2^ + 3.0953*P*] where *P* = (*F*_o_^2^ + 2*F*_c_^2^)/3
2748 reflections	(Δ/σ)_max_ < 0.001
142 parameters	Δρ_max_ = 0.32 e Å^−^^3^
0 restraints	Δρ_min_ = −0.35 e Å^−^^3^

### Fractional atomic coordinates and isotropic or equivalent isotropic displacement parameters (Å^2^)

**Table:**

	*x*	*y*	*z*	*U*_iso_*/*U*_eq_	
S1	0.01389 (4)	0.40662 (4)	0.20839 (10)	0.0345 (2)	
N1	0.13486 (13)	0.28819 (14)	0.2293 (3)	0.0299 (5)	
H71	0.1533 (19)	0.2420 (19)	0.252 (4)	0.045*	
H72	0.1093 (19)	0.3055 (19)	0.312 (5)	0.045*	
C1	0.10172 (14)	0.29629 (14)	0.0560 (3)	0.0227 (5)	
C2	0.04739 (14)	0.35093 (14)	0.0231 (3)	0.0258 (5)	
C3	0.01567 (16)	0.35781 (16)	−0.1524 (4)	0.0342 (6)	
H3	−0.0216	0.3947	−0.1737	0.041*	
C4	0.03771 (17)	0.31167 (17)	−0.2956 (4)	0.0379 (7)	
H4	0.0154	0.3163	−0.4146	0.045*	
C5	0.09245 (17)	0.25864 (16)	−0.2644 (4)	0.0338 (6)	
H5	0.1079	0.2268	−0.3627	0.041*	
C6	0.12488 (15)	0.25144 (14)	−0.0917 (4)	0.0282 (6)	
H6	0.1634	0.2156	−0.0731	0.034*	
C7	0.08960 (15)	0.46865 (15)	0.2526 (4)	0.0301 (6)	
C8	0.10088 (18)	0.49272 (16)	0.4335 (4)	0.0373 (7)	
H8	0.0718	0.4723	0.5315	0.045*	
C9	0.15445 (19)	0.54640 (18)	0.4708 (5)	0.0461 (8)	
H9	0.1613	0.5628	0.5949	0.055*	
C10	0.19821 (19)	0.57669 (17)	0.3324 (5)	0.0485 (9)	
C11	0.18758 (19)	0.55109 (18)	0.1530 (5)	0.0489 (8)	
H11	0.2179	0.5703	0.0560	0.059*	
C12	0.13358 (17)	0.49803 (17)	0.1122 (4)	0.0395 (7)	
H12	0.1267	0.4818	−0.0120	0.047*	
C13	0.2567 (2)	0.6356 (2)	0.3744 (7)	0.0757 (13)	
H13A	0.2811	0.6510	0.2590	0.114*	
H13B	0.2941	0.6149	0.4593	0.114*	
H13C	0.2327	0.6790	0.4322	0.114*	

### Atomic displacement parameters (Å^2^)

**Table:**

	*U*^11^	*U*^22^	*U*^33^	*U*^12^	*U*^13^	*U*^23^
S1	0.0299 (4)	0.0405 (4)	0.0331 (3)	0.0092 (3)	0.0032 (3)	−0.0088 (3)
N1	0.0299 (12)	0.0312 (13)	0.0286 (11)	0.0066 (10)	−0.0038 (10)	−0.0012 (10)
C1	0.0211 (12)	0.0207 (12)	0.0263 (11)	−0.0032 (10)	0.0006 (10)	0.0011 (10)
C2	0.0253 (13)	0.0272 (13)	0.0250 (11)	0.0017 (11)	0.0019 (10)	−0.0025 (10)
C3	0.0386 (16)	0.0352 (16)	0.0287 (12)	0.0081 (13)	−0.0034 (12)	0.0034 (12)
C4	0.0466 (18)	0.0436 (17)	0.0234 (12)	−0.0021 (14)	−0.0029 (13)	−0.0006 (12)
C5	0.0409 (16)	0.0325 (15)	0.0281 (12)	−0.0056 (13)	0.0065 (12)	−0.0050 (11)
C6	0.0270 (14)	0.0227 (13)	0.0349 (13)	0.0008 (11)	0.0062 (11)	−0.0016 (11)
C7	0.0281 (14)	0.0263 (13)	0.0361 (14)	0.0114 (11)	−0.0007 (11)	−0.0040 (11)
C8	0.0436 (18)	0.0323 (16)	0.0360 (14)	0.0102 (13)	−0.0030 (13)	−0.0023 (12)
C9	0.054 (2)	0.0353 (17)	0.0494 (18)	0.0076 (16)	−0.0109 (16)	−0.0085 (15)
C10	0.0423 (18)	0.0264 (16)	0.077 (2)	0.0051 (14)	−0.0024 (18)	−0.0061 (16)
C11	0.047 (2)	0.0338 (17)	0.066 (2)	0.0060 (15)	0.0165 (17)	0.0000 (16)
C12	0.0415 (17)	0.0362 (16)	0.0408 (15)	0.0134 (14)	0.0094 (13)	−0.0021 (13)
C13	0.068 (3)	0.042 (2)	0.117 (4)	−0.0092 (19)	−0.005 (3)	−0.008 (2)

### Geometric parameters (Å, °)

**Table:**

S1—C2	1.771 (3)	C7—C12	1.386 (4)
S1—C7	1.780 (3)	C7—C8	1.389 (4)
N1—C1	1.391 (3)	C8—C9	1.383 (4)
N1—H71	0.90 (3)	C8—H8	0.9500
N1—H72	0.81 (3)	C9—C10	1.379 (5)
C1—C6	1.396 (3)	C9—H9	0.9500
C1—C2	1.399 (4)	C10—C11	1.386 (5)
C2—C3	1.392 (3)	C10—C13	1.516 (5)
C3—C4	1.380 (4)	C11—C12	1.386 (5)
C3—H3	0.9500	C11—H11	0.9500
C4—C5	1.382 (4)	C12—H12	0.9500
C4—H4	0.9500	C13—H13A	0.9800
C5—C6	1.380 (4)	C13—H13B	0.9800
C5—H5	0.9500	C13—H13C	0.9800
C6—H6	0.9500		
			
C2—S1—C7	103.21 (12)	C12—C7—S1	122.5 (2)
C1—N1—H71	114 (2)	C8—C7—S1	118.2 (2)
C1—N1—H72	112 (2)	C9—C8—C7	119.9 (3)
H71—N1—H72	115 (3)	C9—C8—H8	120.1
N1—C1—C6	120.0 (2)	C7—C8—H8	120.1
N1—C1—C2	121.4 (2)	C10—C9—C8	121.7 (3)
C6—C1—C2	118.6 (2)	C10—C9—H9	119.2
C3—C2—C1	119.9 (2)	C8—C9—H9	119.2
C3—C2—S1	119.9 (2)	C9—C10—C11	117.9 (3)
C1—C2—S1	120.00 (19)	C9—C10—C13	121.3 (4)
C4—C3—C2	120.7 (3)	C11—C10—C13	120.8 (4)
C4—C3—H3	119.6	C10—C11—C12	121.3 (3)
C2—C3—H3	119.6	C10—C11—H11	119.3
C3—C4—C5	119.4 (3)	C12—C11—H11	119.3
C3—C4—H4	120.3	C7—C12—C11	120.0 (3)
C5—C4—H4	120.3	C7—C12—H12	120.0
C6—C5—C4	120.6 (2)	C11—C12—H12	120.0
C6—C5—H5	119.7	C10—C13—H13A	109.5
C4—C5—H5	119.7	C10—C13—H13B	109.5
C5—C6—C1	120.7 (2)	H13A—C13—H13B	109.5
C5—C6—H6	119.7	C10—C13—H13C	109.5
C1—C6—H6	119.7	H13A—C13—H13C	109.5
C12—C7—C8	119.1 (3)	H13B—C13—H13C	109.5
			
N1—C1—C2—C3	179.6 (2)	C2—S1—C7—C12	37.3 (3)
C6—C1—C2—C3	2.3 (4)	C2—S1—C7—C8	−147.8 (2)
N1—C1—C2—S1	−5.1 (3)	C12—C7—C8—C9	1.3 (4)
C6—C1—C2—S1	177.62 (19)	S1—C7—C8—C9	−173.8 (2)
C7—S1—C2—C3	−111.1 (2)	C7—C8—C9—C10	−0.7 (5)
C7—S1—C2—C1	73.6 (2)	C8—C9—C10—C11	−0.7 (5)
C1—C2—C3—C4	−0.6 (4)	C8—C9—C10—C13	179.9 (3)
S1—C2—C3—C4	−175.9 (2)	C9—C10—C11—C12	1.5 (5)
C2—C3—C4—C5	−0.7 (4)	C13—C10—C11—C12	−179.1 (3)
C3—C4—C5—C6	0.2 (4)	C8—C7—C12—C11	−0.5 (4)
C4—C5—C6—C1	1.6 (4)	S1—C7—C12—C11	174.4 (2)
N1—C1—C6—C5	179.8 (2)	C10—C11—C12—C7	−0.9 (5)
C2—C1—C6—C5	−2.9 (4)		

### Hydrogen-bond geometry (Å, °)

**Table:**

*D*—H···*A*	*D*—H	H···*A*	*D*···*A*	*D*—H···*A*
N1—H71···N1^i^	0.90 (3)	2.19 (4)	3.083 (3)	170 (3)
N1—H72···S1	0.81 (3)	2.60 (3)	3.032 (3)	115 (3)

Symmetry codes: (i) −*y*+1/2, *x*, −*z*+1/2.
